# PPAR-Gamma Orchestrates EMT, AGE, and Cellular Senescence Pathways in Colonic Epithelium and Restrains the Progression of IBDs

**DOI:** 10.3390/ijms24108952

**Published:** 2023-05-18

**Authors:** Simona Pompili, Antonella Vetuschi, Giovanni Latella, Amarildo Smakaj, Roberta Sferra, Alfredo Cappariello

**Affiliations:** 1 Department of Biotechnological and Applied Clinical Sciences, University of L’Aquila, 67100 L’Aquila, Italy; simona.pompili@univaq.it (S.P.); antonella.vetuschi@univaq.it (A.V.); roberta.sferra@univaq.it (R.S.); 2 Department of Life, Health, and Environmental Sciences, University of L’Aquila, 67100 L’Aquila, Italy; giovanni.latella@univaq.it; 3 Department of Geriatrics and Ortopaedic Sciences, University Cattolica del Sacro Cuore, 00168 Rome, Italy; amarildo.smakaj@gmail.com

**Keywords:** IBD, intestine, inflammation, fibrosis, senescence, β-galactosidase, AGE, RAGE, EMT, PPAR-γ

## Abstract

Intestinal fibrosis, the most common complication of inflammatory bowel disease (IBD), is characterized by an uncontrolled deposition of extracellular matrix proteins leading to complications resolvable only with surgery. Transforming growth factor is the key player in the epithelial-mesenchymal transition (EMT) and fibrogenesis process, and some molecules modulating its activity, including peroxisome proliferator-activated receptor (PPAR)-γ and its agonists, exert a promising antifibrotic action. The purpose of this study is to evaluate the contribution of signaling other than EMT, such as the AGE/RAGE (advanced glycation end products/receptor of AGEs) and the senescence pathways, in the etiopathogenesis of IBD. We used human biopsies from control and IBD patients, and we used a mouse model of colitis induced by dextran-sodium-sulfate (DSS), without/with treatments with GED (PPAR-gamma-agonist), or 5-aminosalicylic acid (5-ASA), a reference drug for IBD treatment. In patients, we found an increase in EMT markers, AGE/RAGE, and senescence signaling activation compared to controls. Consistently, we found the overexpression of the same pathways in DSS-treated mice. Surprisingly, the GED reduced all the pro-fibrotic pathways, in some circumstances more efficiently than 5-ASA. Results suggest that IBD patients could benefit from a combined pharmacological treatment targeting simultaneously different pathways involved in pro-fibrotic signals. In this scenario, PPAR-gamma activation could be a suitable strategy to alleviate the signs and symptoms of IBD and also its progression.

## 1. Introduction

Inflammatory bowel diseases (IBD) represent a wide range of chronic inflammatory disorders of the gastrointestinal tract, including ulcerative colitis (UC) and Crohn’s disease (CD). In approximately 30% of CD patients and almost 5% of UC patients, chronic inflammation leads to intestinal fibrosis [1,2,3]. Intestinal fibrosis is a process characterized by an uncontrolled production and deposition of extracellular matrix (ECM) proteins by activated myofibroblasts in the intestine derived from both resident mesenchymal cells (fibroblasts, sub-epithelial myofibroblasts, and smooth muscle cells) and epithelial and endothelial cells through the process of epithelial–mesenchymal transition (EMT)/endothelial–mesenchymal transition (EndoMT) [4]. Furthermore, the activation of ECM-producing cells is mediated by a plethora of molecules such as cytokines, chemokines, and angiogenic and growth factors. Among these, a key role is played by transforming growth factor-β (TGF-β), which acts via both its canonical (mitogen-activated protein kinase, MAPK; phosphoinositide 3-kinase, PI3K; and suppressor of mothers against decapentaplegic, smad), and non-canonical (i.e., sphingosine 1 phosphate) pathways to promote fibrogenesis onset and progression [5,6,7,8]. Although different molecules, such as glycogen synthase kinase-3 beta (GSK-3β), peroxisome proliferator-activated-receptor gamma (PPAR-γ), and miR200, are able to inhibit TGF-β1-induced fibrosis, to date, no efficient and well-tolerated anti-fibrotic drugs are available [9]. Nowadays, the main clinical options for IBD are drugs aimed at targeting inflammation, such as steroids, salicylates (i.e., sulfasalazine), and biological agents against tumor necrosis factor-α (i.e., infliximab). Indeed, once ECM deposition has begun, it inevitably leads to strictures, stenosis, intestinal obstruction, and consequently a loss of function of the affected digestive tract, and surgery remains the only therapeutic solution [5,6,9,10,11]. IBD has been extensively investigated, and many steps forward have been made over the years. However, the precise etiology of these disorders remains unknown. Several factors are involved in IBD pathogenesis, such as environmental and immunological factors, genetic predisposition, and diet. In this context, over the last two decades, the prevalence of IBD has been increasing constantly and is becoming a global health problem, mainly in the newly industrialized countries (i.e., Brazil and Taiwan) [12,13]. The increased incidence of IBD seems directly correlated with the overconsumption of a Western diet, characterized by high sugar and fat content. These compounds, in response to both industrial (i.e., sterilization) and home-made (i.e., baking) heat treatments, are subject to chemical reaction and reduced into new products such as Amadori products, melanoidins, and advanced glycosylation end products (AGEs) [14,15,16].

AGEs are heterogeneous substances formed by irreversible non-enzymatic interactions between reducing sugars and proteins, lipids, or nucleic acids, a process known as glycation. AGEs can be exogenously introduced or endogenously synthesized in the body. A plethora of classes of exogenous AGEs result from cooking at high temperatures for an extended time, low hydration, and high pH. Endogenous AGEs are mainly produced via the complex Maillard reaction, resulting in the formation of reactive carbonyl compounds. Studies revealed that AGEs, through binding to its receptor (receptor for advanced glycation end products, RAGE), exert a negative effect on human health [17,18,19]. Physiologically, RAGE is slightly expressed in most of the organs, while under pathological conditions, including diabetes, neurodegenerative disorders, and inflammatory diseases, its levels significantly increase [20]. Generally, the fibrotic process is accompanied by upregulation of RAGE, but some exceptions occur, such as lung fibrosis characterized by the loss of RAGE expression [21,22]. Once activated, RAGE is involved in many processes such as inflammation, apoptosis, autophagy, oxidative stress, and cellular senescence.

Cellular senescence is a process characterized by a gradual loss of cell differentiation, loss of nuclear structure and function via downregulation of laminin B1, blockage of cell replication in the G1/S phase, and expression of β-galactosidase resulting in a permanent growth arrest [23]. In response to dangerous stimuli (i.e., oxidative stress, epigenomic dysregulation), a cell can become senescent, activating an apoptotic resistance program and releasing several molecules, including chemokines, interleukins, and matrix proteases, collectively known as senescence-associated secretory phenotype (SASP). This complex secretum alerts neighboring cells to the occurrence of an injury and recruits the immune cells to delete the senescent cells [24]. This scenario preserves the tissue to further exposure to injury and restores tissue fitness and function. However, a prolonged exposure to SASP contributes to the accumulation of senescent cells and the propagation of harmful stimuli and tissue detriment. This situation concurs with the progression of many diseases, especially degenerative ones, such as obesity, cardiovascular disease, steatohepatitis, as well as IBDs [25,26,27,28].

We previously reported that the outcome of DSS-induced colitis is reversed by GED 0507-34 Levo (GED, an agonist of PPAR-γ), showing not only anti-inflammatory activity but also higher effectiveness in fibrosis resolution compared to 5-aminosalicylic acid (5-ASA), the gold standard for IBD treatment. GED was demonstrated to be effective in alleviating the macroscopic and microscopic typical hallmark of DSS-induced IBD, including disruption of organ morphology, reduction in and alteration of crypt architecture, diffuse signs of inflammation in the mucosa and submucosa layers, collagen deposition in the mucosa, submucosa and serosa, and induction of EMT, in in vitro and in vivo models [29,30].

On these bases, EMT, AGEs, and senescence are supposed to be strictly involved with the IBD progression, having in common several molecular players, such as TGF-β and IL-1β. Thus, we aimed to investigate how the EMT, AGEs, and senescence pathways cooperate in the context of the intestinal inflammation and fibrosis process occurring in IBD.

## 2. Results

### 2.1. Human IBD Shows the Activation of EMT, AGE/RAGE, and Senescence

**Figure 1:**
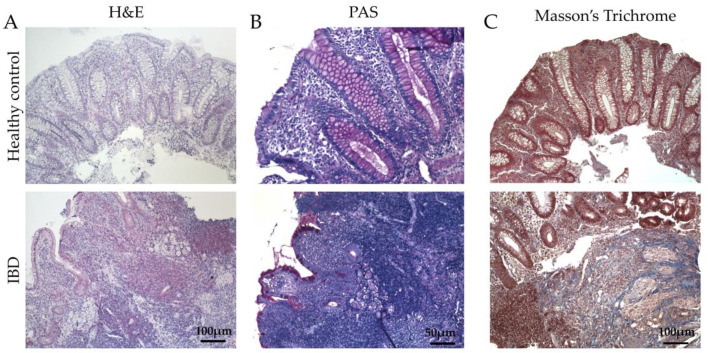
Evaluation of colonic mucosa morphology of IBD patients. (**A**) Hematoxylin and eosin staining to evaluate inflammation. Original magnification 10×, scale bar 100 μm (**B**) Periodic acidic (PAS) staining to evaluate glands morphology. In the control group, the glands appeared intensively colored in magenta red, indicating the presence of a high glycoprotein content. Original magnification 20×, scale bar 50 μm. (**C**) Masson’s Trichrome staining to evaluate fibrosis and organ architecture. Original magnification 10×, scale bar 100 μm. These images are representative of at least n = 4 patients/groups.

To estimate the clinical relevance of our hypothesis, we investigated human biopsies of the colon from healthy subjects and IBD patients in the remission phase of the disease (Figure 1). For one patient, we had biopsies both on healthy and inflamed colonic mucosa (red symbols in the next graphs). First of all, we conducted a morphological analysis to confirm the typical signs of IBD, such as the presence of plain inflammatory infiltrate in the mucosa layer (Figure 1A) as well as glandular atrophy, distortion, and depletion, accompanied by reduced intraluminal secretion (Figure 1B). Finally, signs of fibrosis were highlighted (Figure 1C). In the IBD group, we found a thickness of the extracellular matrix with abundant deposition of collagen fibers in the lamina propria, especially around the glandular residues, compared to control patients (Figure 1C).

**Figure 2:**
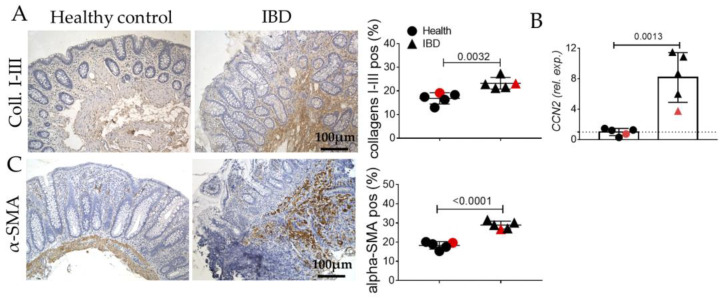
Evaluation of pro-fibrotic signaling (collagens I-III, CCN2, α-SMA), in the colonic mucosa of IBD patients. (**A**) Immunohistochemistry and semi-quantitative analyses for collagens I-III (brown). (**B**) Quantitative Real-Time PCR for Cellular Communication Network Factor 2 (*CCN2*) expression. (**C**) Immunohistochemistry and semi-quantitative analyses for α-SMA. Nuclei are counterstained with hematoxylin; Original magnification 10×, scale bar 100 μm. These images are representative of at least n = 4 subjects/groups. Data were analyzed by unpaired *t*-test. IBD: inflammatory bowel diseases group. Red symbols indicated biopsies from healthy (red circle) and affected (red triangle) colonic mucosa of the same subjects.

Fibrogenesis is confirmed by the high positiveness of collagens I-III at protein and transcriptomic levels (Figure 2A,B) as well as the increase in α-SMA-positive myofibroblasts (Figure 2C).

**Figure 3:**
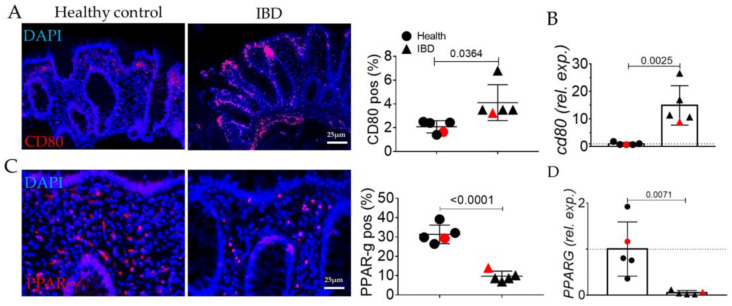
Evaluation of inflammation in the colonic mucosa of IBD patients. (**A**) Immunofluorescence and quantification for CD80 (red). Nuclei are counterstained with DAPI (blue) (**B**) Quantitative Real-Time PCR for *CD80* expression. (**C**) Immunofluorescence and quantification for PPAR-gamma (red). (**D**) Quantitative Real-Time PCR for *PPAR-gamma* expression. Original magnification 20×, scale bar: 25 μm. These images are representative of at least n = 4 subjects/groups. Data were analyzed by unpaired *t*-test. IBD: inflammatory bowel diseases group. Red symbols indicated biopsies from healthy (red circle) and affected (red triangle) colonic mucosa of the same subjects.

Inflammation in the IBD group was confirmed by the evaluation of CD80 (Figure 3A,B). Thus, we analyzed the molecular machinery sustaining morphological alterations. As expected, PPAR-γ is decreased in IBD subjects compared to healthy ones both at the protein and transcriptomic levels (Figure 3C,D).

**Figure 4:**
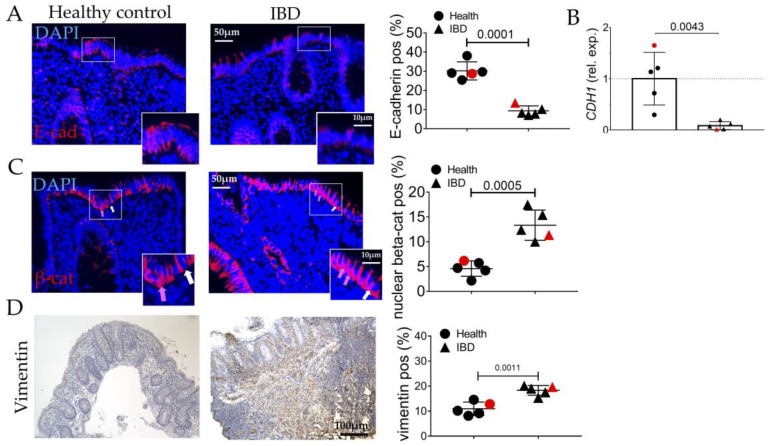
Evaluation of EMT process in the colonic mucosa of IBD patients. *(***A**) Immunofluorescence and quantitative analyses for E-cadherin (red). Nuclei are counterstained with DAPI (blue); Original magnification 20×, scale bar 50 μm. (**B**) Quantitative Real-Time PCR for e-cadherin (*cadh1*) expression. (**C**) Immunofluorescence and quantitative analyses for beta-catenin (red, white arrows) and its nuclear translation (purple, pink arrows). Nuclei are counterstained with DAPI (blue). (**D**) Immunohistochemistry and semi-quantitative analyses for vimentin. Nuclei are counterstained with hematoxylin. These images are representative of at least n = 4 subjects/groups. Data were analyzed by unpaired *t*-test. IBD: inflammatory bowel diseases group. Red symbols indicated biopsies from healthy (red circle) and affected (red triangle) colonic mucosa of the same subjects.

In line with the exacerbated inflammation and fibrosis, EMT was confirmed by the decrease in E-cadherin (Figure 4A,B) and the increase in nuclear translocation of β-catenin (Figure 4C) and in vimentin expression (Figure 4D).

**Figure 5:**
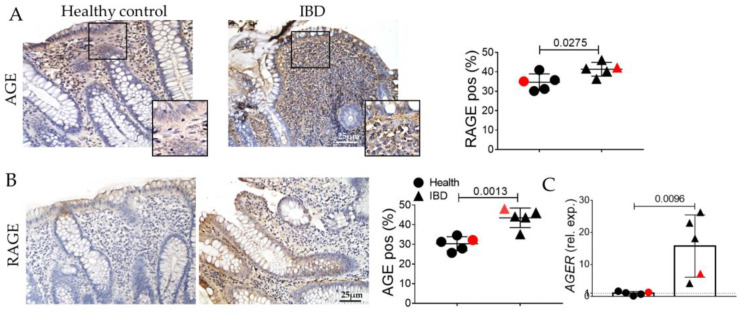
Evaluation of advanced glycosylation end products signaling in the colonic mucosa of IBD patients. (**A**) Immunohistochemistry and quantification for AGE (brown). (**B**) Immunohistochemistry and quantification for RAGE. Nuclei are counterstained with hematoxylin; Original magnification 20×, scale bar: 25 μm. These images are representative of at least n = 4 subjects/groups. Data were analyzed by unpaired *t*-test. IBD: inflammatory bowel diseases group. (**C**) Quantitative Real-Time PCR for age receptor (AGER) expression. Red symbols indicated biopsies from healthy (red circle) and affected (red triangle) colonic mucosa of the same subjects.

Inflammation is reported to upregulate in some circumstances the AGE/RAGE pathway, a crucial player for digestive tract fitness. AGEs are found to be upregulated in patients compared to the healthy group (Figure 5A), and RAGE is concomitantly increased both at the protein and transcriptomic levels (Figure 5B,C).

**Figure 6:**
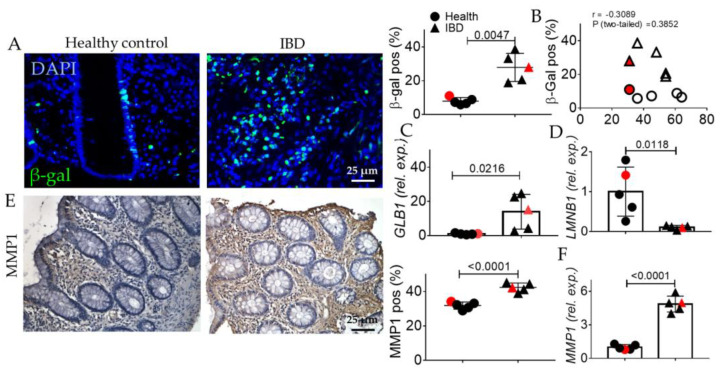
Evaluation of senescent phenotype in the colonic mucosa of IBD patients. (**A**) Immunofluorescence quantification of beta galactosidase (green). Nuclei are counterstained with DAPI (blue). (**B**) Correlation between the age of human subjects and their beta gal positivity. (**C**) Quantitative Real Time PCR for beta galactosidase (galb1) expression. (**D**) Quantitative Real-Time PCR for laminin b1 (*lmnb1*) expression. (**E**) Immunohistochemistry and quantification of beta MMP1 (brown). Nuclei are counterstained with hematoxylin; Original magnification 20×, scale bar: 25 μm. (**F**) Quantitative Real-Time PCR for *MMP1* expression. These images are representative of at least n = 4 subjects/groups. Data were analyzed by unpaired *t*-test. IBD: inflammatory bowel diseases group. Red symbols indicated biopsies from healthy (red circle) and affected (red triangle) colonic mucosa of the same subjects.

Chronic inflammation and activation of RAGE have been reported to be correlated with the activation of senescence. Finally, the expression of acidic beta-galactosidase (β-gal) was assessed. We noted an increase in β-gal positivity in the IBD subjects compared to healthy ones both at the protein and transcriptomic levels (Figure 6A,C), without any correlation between the age of patients and β-gal positivity (Figure 6B). Accordingly, laminin-beta 1 (*Lamnb1*) expression is reduced in the IBD group (Figure 6D). Finally, also the SASP member Matrix MetalloPeptidase (MMP)-1 was increased both at the protein and transcriptomic levels (Figure 6E,F).

### 2.2. GED Reduced EMT Occurring in DSS-Induced Mouse Colitis

**Figure 7:**
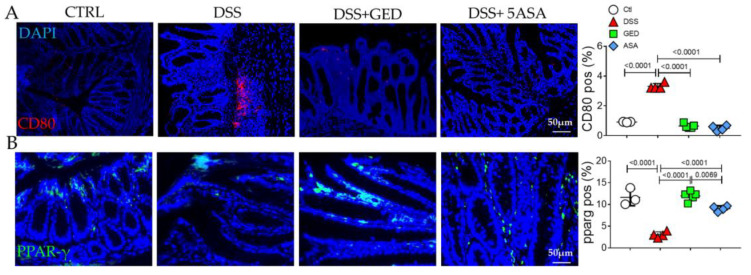
Evaluation of inflammation in the colonic mucosa of DSS-treated mice. (**A**) Immunofluorescence and semi-quantitative analyses for CD80 (red). Nuclei are counterstained with DAPI (blue). (**B**) Immunofluorescence and semi-quantitative analyses for PPAR-gamma (green). Original magnification 20×, scale bar 50 μm. These images are representative of at least n = 3 animals/groups. Data were analyzed by ANOVA (CD80, *p* < 0.0001; pparg, *p* < 0.0001), using Tukey’s test for multiple comparisons (indicated in graphs). Ctl, control; DSS, DSS treated; GED, DSS treated plus GED; ASA, DSS treated plus 5-ASA.

To evaluate the effects of the exacerbated inflammation in intestinal homeostasis in the context of the IBD, we took advantage of the DSS-induced colitis mouse model. According to its anti-inflammatory activity, GED reduced the number of CD80-positive inflammatory cells, similarly to 5-ASA, but activated more efficiently the PPAR-γ signaling in the DSS background (Figure 7A,B).

**Figure 8:**
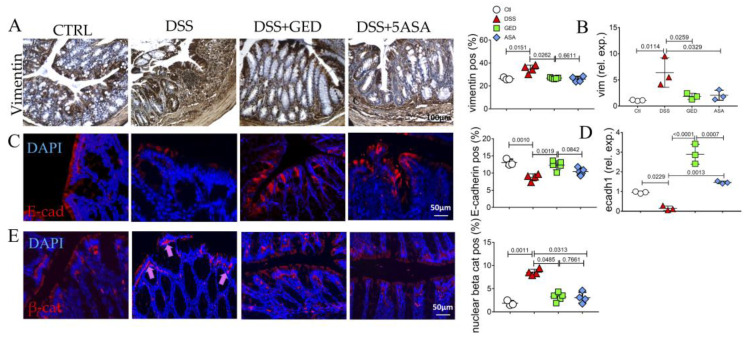
Evaluation of EMT process in the colonic mucosa of DSS-treated mice. (**A**) Immunohistochemistry and quantification for vimentin (brown). Nuclei are counterstained with hematoxylin. Original magnification 10×, scale bar 100 μm. (**B**) Quantitative Real Time PCR for vimentin (vim) expression. (**C**) Immunofluorescence and semi-quantitative analyses for E-cadherin (red). Nuclei are counterstained with DAPI (blue); Original magnification 20×, scale bar 50 μm. (**D**) Quantitative Real-Time PCR for e-cadherin (ecadh1) expression. (**E**) Immunofluorescence and semi-quantitative analyses for beta-catenin (red) and nuclear translation (purple, pink arrows), Nuclei are counterstained with DAPI (blue). These images are representative of at least n = 3 animals/groups. Data were analyzed by ANOVA (vimentin, *p* = 0.0146; vim, *p* = 0.0103; E-cad, *p* = 0.0006; ecadh1, *p* < 0.0001; nuclear β-cat, *p* = 0.0012) using Tukey’s test for multiple comparisons (indicated in graphs). Ctl, control; DSS, DSS treated; GED, DSS treated plus GED; ASA, DSS treated plus 5-ASA.

To evaluate thoroughly the importance of EMT, we compared the regulatory effects of GED and 5-ASA, looking at the expression of the main markers of EMT such as vimentin, E-cadherin, and β-catenin in the colonic mucosa. In line with the exacerbated inflammation and fibrosis, vimentin expression is found to increase in the DSS group (Figure 8A,B). Notably, the modulation of PPAR-γ by GED administration countered the vimentin upregulation (Figure 8A,B). The administration of 5-ASA seems to be less effective in reducing the vimentin expression, validating the presence of fibrosis (Figure 8A,B). E-cadherin was restored at the level of the control group in the mucosa of DSS + GED, according to a more preserved epithelium compared to DSS mice (Figure 8C,D). Finally, a decrease in the nuclear localization of β-catenin was found after GED administration compared to the other groups (Figure 8E). These data confirmed the importance of EMT in IBD progression and the ability of GED to slow down both the fibrotic and EMT process more efficiently than 5-ASA.

### 2.3. DSS-Induced Mouse Colitis Is Accompanied by Advanced Glycosylation End-Products (AGEs) Accumulation, Revertible by GED

**Figure 9:**
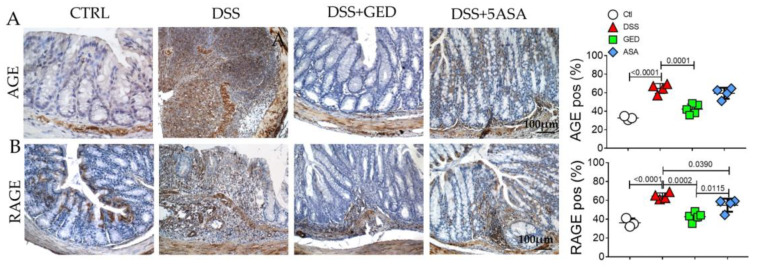
Evaluation of advanced glycosylation end products signaling in the colonic mucosa of DSS-treated mice. (**A**) Immunohistochemistry and semi-quantitative analyses for AGE (brown). (**B**) Immunohistochemistry and semi-quantitative analyses for RAGE. Nuclei are counterstained with hematoxylin; original magnification 10×, scale bar 100 μm. These images are representative of at least n = 3 animals/groups. Data were analyzed by ANOVA (AGE, *p* < 0.0001; RAGE, *p* < 0.0001), using Tukey’s test for multiple comparisons (indicated in graphs). Ctl, control; DSS, DSS treated; GED, DSS treated plus GED; ASA, DSS treated plus 5-ASA.

In this overt background of IBD, we investigated other pathways potentially exacerbating the mucosal disruption as in human samples. As expected, DSS administration increased AGE expression in colonic mucosa (Figure 9A). We noted that the PPAR-γ activation by GED prevented the AGE upregulation induced by DSS, the result also being more efficacious than via 5-ASA, which is not able to mitigate its overexpression (Figure 9A). Accordingly, the RAGE expression (Figure 9B) is reduced by GED, being more effective than 5-ASA.

### 2.4. GED Is Able to Mitigate the Increase in Senescent Phenotype in DSS-Induced Mouse Colitis

**Figure 10:**
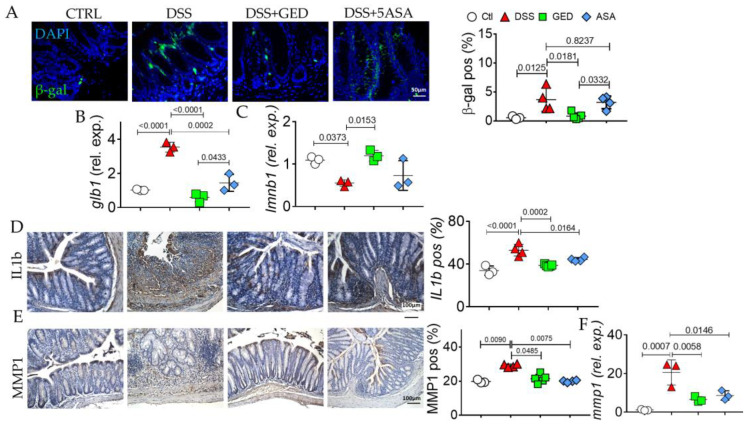
Evaluation of senescent phenotype in the colonic mucosa of DSS-treated mice. (**A**) Immunofluorescence and quantification of beta galactosidase (green). Nuclei are counterstained with DAPI (blue); original magnification 20×, scale bar: 50 μm. (**B**) Quantitative Real Time PCR for beta galactosidase (galb1) expression. (**C**) Quantitative Real-Time PCR for laminin b1 (lmnb1) expression. (**D**) Immunohistochemistry and quantification of beta IL1b (brown). (**E**) Immunohistochemistry and quantification of beta MMP1 (brown). Nuclei are counterstained with hematoxylin. Original magnification 10×, scale bar: 100 μm. (**F**) Quantitative Real-Time PCR for MMP1 expression, n = 3 animals/groups. Data were analyzed by ANOVA (β-gal, *p* = 0.0018; galb1, *p* < 0.0001; lamnb1, *p* = 0.0112; IL1b, *p* < 0.0001; MMP1, *p* = 0.0071; mmp1, *p* < 0.0010), using Tukey’s test for multiple comparisons (indicated in graphs). Ctl, control; DSS, DSS treated; GED, DSS treated plus GED; ASA, DSS treated plus 5-ASA.

**Figure 11:**
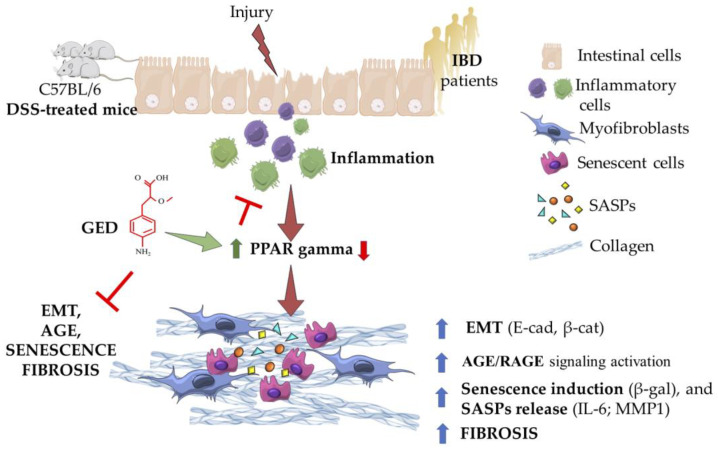
Schematization of intestinal fibrosis occurring in DSS-treated mice and in human IBD. After an injury, intestinal epithelium disruption is induced by inflammation. The persistence of inflammatory response gradually leads to the downregulation of PPAR gamma (red arrow), an abnormal deposition of ECM proteins and the onset of fibrogenesis. EMT, AGE/RAGE signaling, as well as senescence, cooperates with the progression of intestinal fibrosis both in mice and in human (blue arrows). Their activation is mitigated by GED administration in DSS-treated mice, under the control of PPAR gamma (green arrow).

Chronic inflammation and activation of RAGE have been reported to be correlated with the activation of senescence. In line with this, an increase in markers of senescence, β-gal, laminin b1, and SASP members, such as IL-1b and MMP1, were investigated in the DSS group compared to the control (Figure 10). Surprisingly, the activation of PPAR-γ by GED was effective in attenuating the increase in β-gal induced by DSS administration, to a greater extent than via 5-ASA (Figure 10A,B). Interestingly, 5-ASA was unable to rescue the decrease in laminin B1 (Figure 10C), despite the abrogation of some SASP members, namely, IL-1b and IL-6 (Figure 10D–F). We have summarized all the results in Figure 11.

## 3. Discussion

In an attempt to clarify the mechanisms involved in the IBD pathogenesis, in this paper, we focused our attention on the EMT process, the AGE/RAGE signaling as well as their possible correlation with cellular senescence in the context of intestinal fibrosis. Altogether, our data showed that the massive inflammatory status present in patients suffering from IBD and the animal model of DSS-induced colitis is associated with EMT, AGE/RAGE, and senescence activation. Moreover, the administration of the PPAR-γ modulator GED in the animal model of IBD prevented the aberrant activation of all these pathways, endorsing the protective effect of the PPAR-γ on the intestinal mucosa in the context of IBD and widening the role of PPAR-γ as a main orchestrator of the intestinal fitness, being potentially more efficacious than 5-ASA.

AGEs arise from nonenzymatic modifications of proteins by reducing sugars, normally present in the diet and physiologically formed in aging. However, an acceleration of these compounds occurs in oxidative conditions (typical of the inflammation), and their accumulation contributes to the progression of several chronic diseases (i.e., hepatic fibrosis, lung fibrosis, chronic rhinosinusitis, and pelvic organ prolapse), mainly through the activation of the receptor RAGE. Furthermore, AGEs induce EMT and contribute to the stiffening of the extracellular matrix, which alters intracellular communication and induces fibrogenesis [19,31,32,33,34]. Chronic inflammation leads to a process known as “inflammaging”, characterized by accelerated biological aging. Despite senescence being indispensable to limiting the progression of damaged cells, its persistence exerts constant inflammatory effects releasing several proinflammatory cytokines and finally the SASP. In this complex mechanism, AGEs contribute to the inflammatory status, also leading to exacerbation of the senescence and consequently to the release of oxygen free radicals (ROS) and the onset and progression of the inflammation [35,36,37,38].

IBD is a spectrum of diseases, including CD and UC, affecting the gastrointestinal tract. Frequently, these disorders switch from chronic inflammation to intestinal fibrosis, a process characterized by an uncontrolled accumulation of ECM proteins by activated myofibroblasts. Fibrosis leads to a rearrangement of normal tissue architecture, and irreversible complications such as strictures, stenosis, and intestinal obstruction, principally in CD. Currently, no resolutive pharmacological remedies are available for IBD treatment once the ECM deposition has begun. Nowadays, anti-inflammatory drugs, such as sulfasalazine, are the palliative therapeutic choice with mainly anti-inflammatory effects, and surgery remains the only definitive treatment for the severe forms of IBD. Intestinal fibrogenesis is driven by complex mechanisms orchestrated by TGF-β [39]. Moreover, many other TGF-β-agonist pathways concur with the progression of IBD, still poorly understood. For this reason, many preclinical efforts against intestinal fibrosis are oriented towards the use and optimization of promising TGF-β modulators exerting anti-fibrotic action, including PPAR-γ and its natural (i.e., omega 3 and 6) and synthetic agonists (glitazones).

In human biopsies, morphological analyses revealed, as expected, inflammation, gland alterations, and fibrosis in IBD patients compared to the control (Figure 1 and Figure 2). Concurrently, we found an increase in the expression of the inflammatory marker CD80 (Figure 3) as well as in the EMT markers (Figure 4), and AGE/RAGE pathway (Figure 5) in the IBD group, thus validating the activation of fibrotic signaling. Moreover, a senescent process accompanied the fibrosis progression in IBD patients. Indeed, we highlighted an increase in β-gal and MMP1 and a decrease in laminin B1 in the IBD group compared to the control (Figure 6).

To mimic the human IBD, we used an in vivo model of DSS-induced colitis and we found features of inflammation compared to control mice as revealed by CD80 evaluation (Figure 7). Similarly, the expression of the main markers of EMT (Figure 8), and AGE/RAGE signaling members increased in DSS mice (Figure 9), confirming the presence of fibrosis. The same molecules decreased in our PPAR-γ agonists-treated groups, both in 5-ASA and GED (Figure 7). Furthermore, to support our hypothesis of senescence involvement, we found an increase in β-gal and SASP (Figure 10) expression in the DSS group, confirming the presence of a senescent phenotype correlated with fibrosis. Surprisingly, via GED administration, it is possible to reduce senescence with greater efficacy than via 5-ASA treatment (Figure 10A,B). We believe that our data could represent a step forward in the knowledge of the complex IBD world. Indeed, TGF-β is an inducer of EMT, and ECM deposition is supported by AGEs, which are in turn introduced into the body with food. Finally, it is known that both TGF-β and RAGE can accelerate the cellular senescence process, as they encourage the production of oxygen free radicals (ROS) and increase oxidative stress. Furthermore, all these biological processes are crucial in the chronicity of inflammatory states, as they are involved in the activation of immune cells.

Overall, our observations indicate that the IBD is sustained by the activation of multiple pathways having detrimental effects on intestinal fitness: inflammation, fibrosis, and activation of EMT, AGE/RAGE and senescence pathways. Our results are in line with other papers. In fact, CD80 overexpression is reported both in human and mouse IBDs [40,41,42]. Accordingly, the AGE/RAGE axis is reported as a key driver of IBD, and multiple beneficial effects are reported for PPAR-gamma agonists against metabolic syndromes and age-related diseases [43,44,45,46]. In addition, senescence is associated with the progression of intestinal inflammation and illnesses, such as colorectal cancer, and senolytics are promising therapies for degenerative diseases [47,48].

All of the previous pathways establish a positive feedback loop, leading to intestinal damage and organ failure. We highlighted that this background is present in patients suffering from IBD in the remission phase of the disease. The remission phase is clinically reached when the disease is plenty asymptomatic and healthy by mucosal endoscopy. Unfortunately, endoscopic mucosal healing does not fit always with histological healing, and patients frequently experience recurrence of the acute phase. Interestingly, although the patients are under pharmacological treatments and follow a moderately healthy lifestyle to prevent the acutization of the disease, the AGE/RAGE axis is still exacerbated, suggesting an endogenous and/or idiopathic alteration rather than a triggering from the diet. Furthermore, our data indicate that IBD patients can take advantage of a combination therapy that can trigger the PPAR-γ pathway and inhibit the AGE/RAGE and senescence axes. Notably, this pathogenetic loop can be attenuated in the animal model using a PPAR-γ modulator, GED, endorsing the protective role of PPAR-γ in colon homeostasis and the use of its modulator for the treatment of patients suffering from IBD.

## 4. Materials and Methods

### 4.1. Animal Experiments

This study used samples recovered from a previous study performed at the Institution of Pasteur Animal care facility (Institut Pasteur de Lille, Lille, France) according to governmental guidelines and approved by the “Comité d’Ethique en Expérimentation Animale Nord-Pas de Calais” (CEEA n°75; ethics committee for animal experimentation of the region Nord-Pas de Calais, France). All animals were housed in plastic cages and kept in a pathogen-free environment, under constant room temperature, with a 12 h/12 h light/dark cycle. The animals were fed a standard diet and had free access to water. Sixteen male C57BL/6 mice (Janvier, Le Genest-St-Isle, France) were included in the present study.

### 4.2. Induction of Chronic Colitis, Drugs, and Experimental Design

The mice were randomly divided into four groups: control (H_2_O) n = 3, DSS n = 4, DSS + GED n = 5, and DSS + 5ASA n = 4. Chronic colitis and fibrosis were induced in mice by oral administration of 2.5% (*w*/*v*) DSS (MW: 36,000–44,000, purchased from TdB Consultancy, Uppsala, Sweden), solubilized in autoclaved tap water and administered ad libitum for three cycles (5 days DSS, followed by 7 days of tap water). The control group received only tap water. GED-0507-34 Levo (Nogra Pharma Ltd., Dublin, Ireland), a selective agonist of PPAR-γ, was dissolved in a solution containing 0.5% Carboxymethylcellulose sodium salt (Sigma-Aldrich, Darmstadt, Germany) and 1% Tween 80, administrated by oral gavage (100 μL/mouse), at a dose of 30 mg/Kg/day. The 5-aminosalicylic acid (5-ASA) (Pentasa, Ferring Pharmaceuticals, Gentilly, France), the most common anti-inflammatory drug used in IBD treatment, was mixed with standard chows and was administrated daily at a dose of 150 mg/kg. All drugs were administrated at the beginning of the second cycle of DSS (day 12).

### 4.3. Sample Recovery and Preparation

The animals of each group were sacrificed four days after the last DSS cycle administration. Following laparotomy, colon was identified and rapidly excised. Then, the colorectal samples were subjected to conventional histological processing procedures, fixation in 4% buffered formalin in phosphate-buffered saline (PBS) (pH 7.4 for 3 h at room temperature), and paraffin embedding.

### 4.4. Human Biopsies: Patients and Methods

In this study, we examined a total of nine patients recruited at the San Salvatore Hospital at L’Aquila by the Gastroenterology unit and dived into two groups: the control group (n. 4) and the clinical case group (n. 5). Control group included people who underwent coloscopy for in-depth diagnostic analysis given the presence of a familiarity history of colorectal cancer, rectorrhages, abdominal pain correlated to the irregularity of the hive, presence of occult blood in the stool associated or not with anemia, and follow-up of patients subject to previous endoscopic removal of adenomatous colon polyps. The clinical cases group included patients suffering from IBD, including both Crohn’s disease and ulcerative colitis, who underwent control coloscopy to assess the severity of the intestinal lesions. Both controls and patients included in the study were over 18 years old, the control group having a median age of 51.5 years (range 36–64) and the IBD group a median age of 48 (range 31–54). The Institutional Ethics Committee of University of L’Aquila (prot. n. 46004; 29 November 2017) approved the investigation protocol, and all eligible patients signed a consent form for the processing of personal data and to allow the excision of tissue and its use for this study.

### 4.5. Histomorphological Analysis

Serial 3 μm sections were stained using Hematoxylin and Eosin (H&E) (Bio Optica, Milano, Italy), in order to highlight the degree of inflammation, and Masson’s Trichrome (Bio Optica, Milano, Italy), to evaluate the deposition of connective tissue and fibrosis. Furthermore, human samples were stained with Periodic acid-Schiff (PAS, Bio Optica, Milano, Italy) to assess changes in the number of goblet cells. The stained sections were then observed under an Olympus BX51 Light Microscope (Olympus Optical Co., Ltd., Tokyo, Japan). Quantification of markers in mouse sections was performed on three whole sections and normalized against the total section area, while for human samples, four fields were randomly analyzed and normalized against the number of nuclei. Two independent pathologists performed blind evaluations.

### 4.6. Senescence Assay

The presence of active endogenous acidic beta-galactosidase was revealed by CellEvent™ Senescence Green Assay Kit (Thermo Fisher Scientific, Waltham, MA USA), in accordance with the manufacturer instructions.

### 4.7. RNA Extraction and Real Time-PCR

Total RNA was extracted using deparaffinization solution (Qiagen, cat. QG19093, Hilden, Germany) and miRNeasy FFPE Kit (Qiagen, cat QG217504) according to manufacturer’s instruction from 5 sections (10 µm each) of FFPE (formalin fixed paraffin embedded) samples of murine and human colons. Then, RNA (0.8 μg) was reverse transcribed via Reliance Select cDNA Synthesis KT (Bio-rad, cat. 12012801, Hercules, CA, USA). cDNA was subjected to real-time PCR, using the iQ™ Multiplex Powermix, (Bio-rad, cat. 1725849), together with gene-specific PrimePCR™ Probe Assays (Bio-rad, Table 1) for detecting expression of human genes or using PowerUp SYBR Master Mix (TermoFisher) with gene-specific primers (Themo fisher, Table 2) for detecting expression of mouse genes. Reactions were set up in Primo^®^ FrameStar^®^ 96-well PCR plates (Euroclone, cat. ECPCR0770C, Pero, Italy), which were sealed with MicroAmp™ optical adhesive films (Applied Biosystems, cat. 4360954, Waltham, MA, USA). The thermal profile of Cielo 6 qPCR system (Azure Biosystem, Dublin, CA, USA) was set as follows: 2 min at 95 °C, then 45 cycles of 15 s at 95 °C and 20 s at 60 °C, along with a final post-read stage of 30 s at 60 °C.

### 4.8. Immunohistochemistry and Immunofluorescence

Colonic samples were also promptly fixed in 4% buffered formalin in PBS for 3 h at room temperature, dehydrated in graded ethanols and embedded in low-melting paraffin. Sections of 3 μm in thickness were incubated in methanol and in 3% hydrogen peroxide solution for 15 min. The specimens were incubated overnight at 4 °C with specific antibodies. The samples were washed in PBS for 5 min and finally incubated for 1 h at room temperature with the appropriate secondary antibody, horseradish (HRP) (EnVision^®^ + Dual Link System-HRP (DAB+); Agilent, Santa Clara, CA, USA) or fluorophore conjugated. Finally, for IHC, the sections were counterstained with Mayer’s hematoxylin and mounted with Eukitt medium, while for IF, the sections were mounted with Fluorlast with DAPI medium for nuclear counterstaining (Biovision, Milpitas, CA, USA). The specimens were observed under an Olympus BX51 light microscope equipped with a laser source (Olympus, Optical Co., Ltd.).

### 4.9. Semiquantitative Digital Image Analysis of Immunohistochemical Staining

Semiquantitative evaluation of immunohistochemical staining was performed using the immunohistochemistry profiler, a plugin of a digital image analysis public domain software ImageJ version 1.52a (U.S. National Institutes of Health, Bethesda, MD, USA) [49,50]. Four random microscopic fields from all experimental groups were photographed at the same magnification and analyzed. Data were expressed as a percentage of the positive area and presented as mean ± standard deviation. Statistical significance was set at *p* ≤ 0.05.

### 4.10. Statistical Analyses

Results were expressed as mean ± standard deviation. Statistics were run using ANOVA with unpaired non-parametric Mann–Whitney test, which was used for statistical analyses among more than two experimental groups; a *p*-value < 0.05 was considered statistically significant. All experiments involving animals were performed using at least 3 mice/group. Statistics between two experimental groups involving human subjects (at least 4 individuals/group) were run using an unpaired *t*-test, and a *p*-value < 0.05 was considered statistically significant. (GraphPad Prism 7.00; GraphPad Software, Inc., La Jolla, CA, USA).

## Figures and Tables

**Table 1:** List of probes used for human gene expression analysis by Taqman assay.

PrimerPCR Probe Assay, Fluorophore	Unique Assay ID
*Actb,* HEX	qHsaCEP0036280
*Ager,* FAM	qHsaCEP0040022
*Ccn2,* TEX615	qHsaCEP0024255
*Cd80,* Cy5.5	qHsaCIP0026764
*Cdh1,* Cy5.5	qHsaCEP0049339
*Glb1,* TEX615	qHsaCEP0057625
*Lmnb1,* FAM	qHsaCIP0029571
*MMP1,* Cy5	qHsaCEP0055366
*Pparg,* Cy5	qHsaCEP0051687

**Table 2:** List of primers used for mouse gene expression analysis by Sybr Green assay.

Gene Name	Sequence 5′-3′
*Actb FW*	CCACCATGTACCCAGGCATT
*Actb RW*	CGGACTCATCGTACTCCTGC
*Cdh1 FW*	AGAATGAGGTCAATGCCCGG
*Cdh1 RW*	TGTATTGCTGCTTGGCCTCA
*Glb1 FW*	CATCTCGGGAAGCATTCATT
*Glb1 RW*	CGGTCCCCAGAAAACTCATA
*Lmnb1 FW*	TGCTGCTCAATTATGCCAAG
*Lmnb1 RW*	TGCTTCTAGCTGGGCAATCT
*MMP1 FW*	GTTGCTTCTCTGGGCTGCTA
*MMP1 RW*	CAGCCATCATCTCCTTGCCA
*Vimentin FW*	GATCAGCTCACCAACGACA
*Vimentin RW*	GGTCAAGACGTGCCAGAGAA

## Data Availability

Data available on request due to restrictions. The data presented in this study are available on request from the corresponding author. The data are not publicly available due to restriction for patients’ privacy.
